# IoT-Driven Pull Scheduling to Avoid Congestion in Human Emergency Evacuation

**DOI:** 10.3390/s26030837

**Published:** 2026-01-27

**Authors:** Erol Gelenbe, Yuting Ma

**Affiliations:** 1 IITiS-PAN, 44100 Gliwice, Poland; yma@iitis.pl; 2 CNRS I3S, Université Côte d’Azur, 06903 Sophia Antipolis, France; 3 Department of Engineering, King’s College London, London WC2R 2LS, UK

**Keywords:** emergency evacuation, Internet of Things, maritime passenger transport, cruise ships, evacuation delay, G-Networks

## Abstract

The efficient and timely management of human evacuation during emergency events is an important area of research where the Internet of Things (IoT) can be of great value. Significant areas of application for optimum evacuation strategies include buildings, sports arenas, cultural venues, such as museums and concert halls, and ships that carry passengers, such as cruise ships. In many cases, the evacuation process is complicated by constraints on space and movement, such as corridors, staircases, and passageways, that can cause congestion and slow the evacuation process. In such circumstances, the Internet of Things (IoT) can be used to sense the presence of evacuees in different locations, to sense hazards and congestion, to assist in making decisions based on sensing to guide the evacuees dynamically in the most effective direction to limit or eliminate congestion and maximize safety, and notify to the passengers the directions they should take or whether they should stop and wait, through signaling with active IoT devices that can include voice and visual indications and signposts. This paper uses an analytical queueing network approach to analyze an emergency evacuation system, and suggests the use of the Pull Policy, which employs the IoT to direct evacuees in a manner that reduces downstream congestion by signalling them to move forward when the preceding evacuees exit the system. The IoT-based Pull Policy is analyzed using a realistic representation of evacuation from an existing commercial cruise ship, with a queueing network model that also allows for a computationally very efficient comparison of different routing rules with wide-ranging variations in speed parameters of each of the individual evacuees.Numerical examples are used to demonstrate its value for the timely evacuation of passengers within the confined space of a cruise ship.

## 1. Introduction

The management of major emergencies to minimize loss of life, maximize human well-being, and minimize loss of property and economic damage is an active and well-established area of research. Such emergencies frequently occur in peacetime, and tragically, also occur during warfare, as we see today [1,2]. Commonly occurring recent examples of emergencies that require rescue and evacuation include the following:

- The frequent fires that occur around the Mediterranean coast, in particular during the summer period [3];
- The annual hurricane season in the southeastern United States, including Florida, that can require the evacuation of millions of people along congested roads [4];
- Seasonal floodings that occur regularly in various parts of the world, including in Europe, such as the floods in northeastern Spain in 2024 [5];
- Accidents or malevolent events that have occurred over recent years in theaters, sports arenas, large hotels, and during cultural or sports events [6,7];
- Accidents involving cruise ships, as discussed in Section 1.3;
- Industrial accidents in factories, building sites, and underground or overground mines [8,9].

Though each event is distinct and different, all these events are characterized by a few common characteristics:

- Physical space is constrained because it is a built environment with buildings and roadways, some of which become blocked during the emergency;
- Civilians have to be evacuated rapidly, while emergency personnel, such as ambulances, police, firemen, and expert volunteers, have to enter the area rapidly;
- The civilian evacuees include both able-bodied people and others who have to be helped or carried out due their age or injuries;
- A portion of the area contains health hazards such as fires, smoke, or high levels of pollution or noxious materials or gases.

Thus, addressing these challenges has often required the use of specialized and complex cyber–physical systems [10], and substantial work has been conducted on developing algorithmic methods to address them [11,12]. A key issue in this area is understanding the performance of the methods that are used to address such emergencies, such as exit times for civilians, expected survival rates, speed with which emergency staff and equipment arrive at the scene, and other metrics, all of which can require sophisticated modeling techniques [13].

The need to address these challenges via a comprehensive modeling approach that attempts to encompass all the significant aspects of an emergency has resulted in a long line of simulation studies [14], and several special-purpose simulation tools have been developed [15]. The highly distributed structure of such events, with a large number of autonomous human and possibly robotic agents interacting with each other and affecting the outcome, has given rise to both agent-based simulators [16] and analytical modeling studies.

### 1.1. Technical Challenges

In addition to the need for realistic simulations, emergency evacuations have several common technical challenges that are worth mentioning. They all require emergency personnel to be able to search the relevant areas to identify hazards and locate victims; thus, some work has sought inspiration from the efficiency with which certain animal species search for food, or for safe areas, as well as from other techniques such as foraging and the behavior of physical systems that identify and locate targets for physical or chemical interactions [17]. Another common aspect is the need to make “good” but not necessarily optimal decisions in a very short time. This requires software-based decision aids and fast and accurate decision algorithms that represent the space, such as in [18]. Since evacuees should be able to move (or be moved) rapidly and efficiently to exit areas or other safe areas where they can receive adequate care, emergency management requires efficient routing algorithms; however, many such algorithms are computationally intensive [19] and may not be adequate for providing decisions in real time with partial or incomplete information [20].

Many of the above considerations underline the need for communication in emergency evacuation systems, where public mobile networks may be “down” due to physical events or a lack of sources of electricity [21]. In such cases, one must seek communication technologies that are self-aware and self-organized and that adapt to possible congestion in the network. Thus, disruption-tolerant and opportunistic delay-tolerant communication systems have been suggested [22].

When communications are discussed in the context of emergency management, one must also consider the issues of cybersecurity, as well as how smart cyberattack detection can be carried out and mitigated, rapidly and effectively. In addition, such cyberattacks can target the security of end users and the signaling schemes that allow the network to establish communications [23].

In addition, any technical support tools, such as communication networks, sensors, actuators, or computational support in the form of local edge computing or more remote Cloud computing, must be readily available and locally implementable to be of any use in rapid decision-making. Such systems must also be compatible with local existing infrastructures and must satisfy the safety and installation-related regulations of the local systems, such as buildings or ships. While all these aspects are important to emergency management systems, in the next Section, we will turn more specifically to the evacuation of cruise ships, which is the subject of this research.

### 1.2. The Cruise Industry

**Figure 1:**
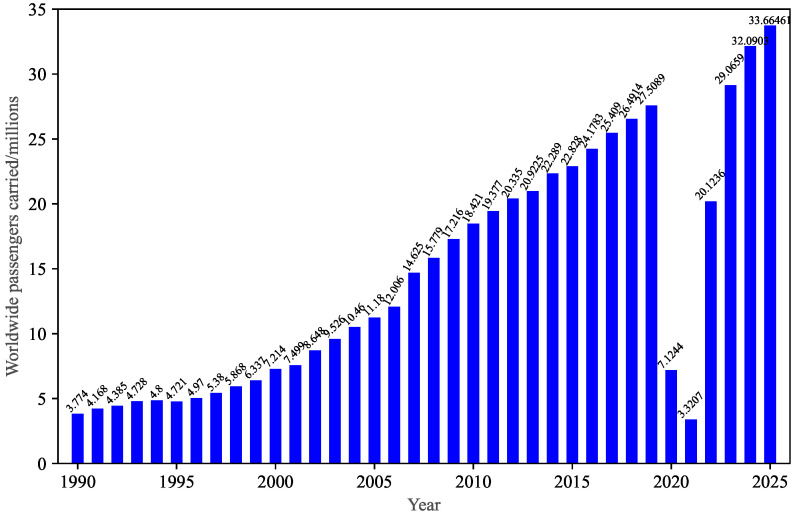
Number of global cruise ship passengers from 1990 to 2025.

**Figure 2:**
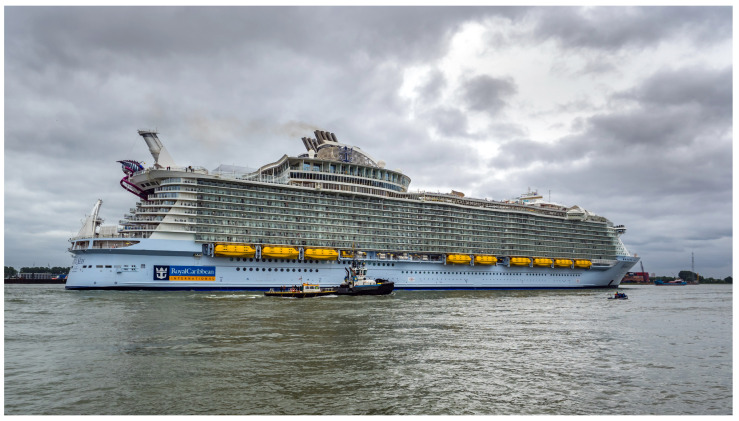
View of the cruise ship Harmony of the Seas.

The cruise industry is a vital component of the global tourism sector, having experienced the fastest growth prior to the COVID-19 pandemic, and contributing significantly to the global economy, job creation, and cultural exchange [24,25]. According to the State of the Cruise Industry Report, 2025, published by Cruise Lines International Association (CLIA), the ocean cruise industry experienced a compound annual growth rate of 6.3% in passenger amounts from 1990 to 2025 [26]. As shown in Figure 1, while the ocean cruise industry was brought to a standstill for nearly two years by the COVID-19 pandemic, it has demonstrated a rapid rebound. By 2023, cruise travel had already reached 106% of the pre-COVID levels of 2019, with 29.1 million passengers sailing, indicating the strong resilience of cruise tourism in the face of global downturns. Simultaneously, fifteen new cruise ships, with a combined passenger capacity of 38,629, are expected to be completed by 2025, increasing the global ocean cruise passenger capacity to 704,200 across 370 vessels [27], with large cruise ships becoming a dominant force in the cruise industry. Such vessels typically feature highly complex internal layouts, comprising multiple decks with restaurants, theaters, shopping areas, pools, and other entertainment venues, and hundreds of passenger cabins (see Figure 2). Although the frequency of large cruise ship accidents has decreased significantly over the last decade owing to the introduction of new safety regulations and guidelines, improved crew training schemes, and technological innovations, the consequences of such accidents remain catastrophic.

The next section collates and analyzes ship accidents that have occurred during this century, with particular focus on the role of evacuation in these accidents, in order to highlight the importance of effective evacuation and to identify key factors to consider when designing emergency evacuation approaches for passengers on vessels.

### 1.3. Marine Casualties and Incidents

**Figure 3:**
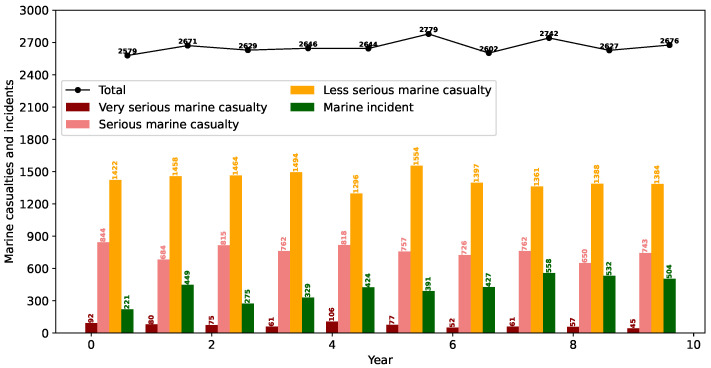
Evolution of the number of marine casualties and incidents over time, presented by order of severity.

Grounding (or stranding), contact, collision, and fire constitute the predominant categories of accidents for passenger ships of all subtypes, including cruise ships, pure passenger ships, and passenger Ro-Ro cargo vessels [28]. Such events generally pose a serious threat to the viability of the ship, crew, passengers, and cargo. According to the Annual Overview of marine casualties and incidents 2024 published by the European Maritime Safety Agency (EMSA), there are a total of 6370 passenger ships involved in marine casualties and incidents in the territorial seas of European Union (EU) member states during the period from 2014 to 2023, resulting in 2149 injuries and 54 fatalities [29]. Among these marine casualties and incidents, 53.4% are classified as less serious, 28.4% as serious, 2.7% as very serious, and 15.5% as marine incidents (see Figure 3). In addition to passenger ship accidents in the territorial seas of EU member states, accidents also occur from time to time in other seas. In 2002, the MV Le Joola RO-RO ferry capsized off the coast of The Gambia, resulting in 1863 fatalities and 64 survivors, ranking as the third-worst peacetime disaster in maritime history [30]. There were several immediate factors playing a role in this disaster, including poor cargo stowage, severe passenger overloading, engine failure, adverse weather, and improper evacuation. With regard to the evacuation process, the excess passengers concentrated on the upper decks, making the ship highly unstable. Moreover, to take cover from the storm, they rushed en masse to one side, which further aggravated the hull tilt and significantly accelerated the capsizing. In 2006, the MS al-Salam Boccaccio 98, a RO-RO passenger ferry, sank in the Red Sea, resulting in over 1000 deaths [31]. The immediate cause of the sinking was identified as the buildup of seawater in the hull during firefighting operations in the engine room, which induced a severe listing and ultimately led to the ship capsizing.

According to the Panama Maritime Authority Preliminary Investigation Report, at 19:09, the fire alarm on MS al-Salam Boccaccio 98 was activated, providing visual and audible alerts on the control panel at the bridge, but orders to evacuate the vessel were never given or carried out as per established procedures. The majority of passengers remained waiting for evacuation instructions from the master until the vessel sank. In 2014, the ferry MV Sewol foundered while en route from Incheon to Jeju in South Korea under calm sea conditions [32]. Out of 476 passengers and crew, only 172 people survived this disaster. The primary cause of the sinking was an unreasonably sudden turn to starboard, which triggered a shift of cargo to port and, in turn, induced a heavy listing and the eventual capsizing. The captain ordered passengers to stay in their cabins rather than mobilizing an evacuation of passengers while the Sewol ferry was listing. Therefore, this accident is considered to be a man-made disaster, since all passengers could have survived if adequate evacuation procedures had been implemented. In 2021, the passenger ferry MV Avijan-10 caught fire on the Sugandha River [33]. This accident resulted in more than 40 deaths and more than 100 injuries. Most of the victims either died from the fire or drowned during their escape. Analysis of the aforementioned ship accidents indicates that the complete elimination of maritime accidents is practically unrealistic and that inappropriate post-accident evacuation is one of the major factors contributing to the associated catastrophic consequences.

Conversely, the implementation of effective evacuation procedures can substantially mitigate maritime accidents in terms of reducing the resultant fatalities and injuries. In 2013, the MV Grandeur of the Seas, carrying 2224 passengers and a crew complement of 796, was on fire [34]. Once the emergency was declared, the crew was first sent to their emergency stations, and passengers were directed to their muster stations. Therefore, despite the serious and large conflagration causing significant structural damage, no personal injuries were reported among passengers or crew members, thanks to the timely and effective emergency evacuation. Similarly, all individuals aboard the Yangtze Grand View No. 7 cruise ship were safely evacuated to shore within 29 min, with no reported injuries. In summary, the evacuation decision plays a crucial role in protecting the lives of those on board when a passenger ship is involved in a serious accident.

Evacuation planning for passengers on a damaged ship is a highly complex transhipment problem, affected by factors such as the propagation of hazards, the spatial and temporal distribution of evacuees and life-saving appliances, the dynamic inclinations of the ship, the capacity of muster stations, and the survival time of the vessel. In addition, the design of evacuation strategies is influenced by the underlying support infrastructure available on board a ship. All cruise ships are required by the International Convention for the Safety of Life at Sea (SOLAS) to pre-deploy static evacuation plans to direct evacuees to muster stations in the event of an emergency. These plans must indicate the current area or cabin of passengers, the designated assembly area, the locations of lifeboats and liferafts, and the escape routes. Static evacuation plans are designed to provide paths with the shortest distance to muster stations, but they neglect other essential factors influencing evacuation efficiency and passenger safety. In recent years, intelligent ships equipped with advanced technologies such as the Internet of Things (IoT) have gradually become the norm in the cruise industry. Various sensor-based evacuation approaches have been proposed to provide dynamic evacuation plans that can react to changing conditions on board and are able to adapt guidance using real-time data collected by sensors.

Therefore, this paper investigates how passenger routing and scheduling strategies can impact the time it takes to evacuate each passenger in the presence of variable passenger walking speeds and congestion due to queueing delays in the narrow staircases and passageways. We also introduce a “Pull Policy,” which can be implemented with the support of an Internet of Things (IoT) network, which uses messaging to synchronize the motion of passengers from waiting areas into the evacuation paths based on exit incidences of previously scheduled passengers, showing that this policy can lower the overall average delay experienced by passengers as they exit the vessel. These results are shown with the use of a mathematical queueing model based on G-Networks [35], with fast and accurate computation of individual and overall delays.

Thus, in Section 2, we first review related work. Then, in Section 3, the analytical modeling approach that we use is detailed. Section 4 discusses the “pacing or regulating” scheme, which we call the “Pull Policy,” to guarantee that congestion does not form in the various parts of the emergency evacuation system. In Section 5, we present the numerical results concerning the Pull Policy, which is a heuristic extension that sends messages simultaneously to all source nodes, together with the different routing rules we have examined. Finally, Section 6 presents our conclusions and suggests future research directions.

## 2. Related Work

Emergency evacuation methods are studied to improve the safety of people, property and vehicles [36], and they increasingly rely on modern Internet of Things (IoT) technologies such as sensing, decision making, and communications. These methods are studied to assist human evacuees and vehicles, locating them and guiding them towards safety by relaying information about ongoing conditions and guiding them along the fastest and safest pathways [37,38]. Such methods can be enhanced with crowd monitoring [39] and the automatic selection of evacuation paths [40]. The study of emergency evacuation faces the obvious difficulty of artificially reproducing emergencies. Thus, modeling and simulation have become essential in this context [41,42] and are routinely used for the design and comparison of appropriate methods and algorithms [43] that improve or optimize the effectiveness and robustness of evacuation procedures.

Modeling and simulation rely on two distinct methodologies:

1. Analytical Modeling (AM), which has long been used for the study of communication, computer, networks and transportation systems [44,45], where systems of equations are used to represent reality.
2. Discrete Event Simulation (DES) [46], where the events in a system are captured in a computer program that represents, in great detail, the sequence of events that unfold over time within the real world that we wish to represent, with probabilistic or imperfectly known transitions being represented by random variables.

While DES can require substantial programming efforts and lengthy computation time, offering the possibility of representing the system very accurately, AM generally offers faster computational algorithms but requires the solution of a mathematical model which may not be readily available or which may be intractable.

Emergency management simulation research addresses the movement of people in certain scenarios, including sports arenas, tourist sites, leisure venues [47,48], and large ships [49,50], in adversarial situations where some of the infrastructure may have broken down and where hazards such as fire and the effect of human panic may further impair the evacuation process, such as in large-scale maritime transport [51,52] and other areas of emergency management [53,54].

On the other hand, emergency management systems are designed to guide evacuees using path-planning or dynamic algorithms, including the grouping of evacuees [55] to minimize evacuation delay and maximize the distance between the safe paths being used and the hazards and congestion which may occur. Such methods use 2D/3D maps of the built or natural environment in which the evacuation is occurring [56] and compute changes in the paths when unexpected congestion and hazards such as fire, flooding, or failures in communication systems occur. Situations where dynamic dangers can exist require techniques for establishing relatively stable evacuation paths, even when unexpected hazards emerge, as has been achieved using the “Expected Number of Oscillations (ENO)” concept [57], which identifies paths with the smallest probability of frequent changes. While global path planning methods can require complete system specifications that are unavailable in realistic scenarios, several recent studies have avoided this requirement by allowing the path computation to proceed without precise prior knowledge of all hazards and without detailed information about all possible exits [58].

The International Maritime Organization (IMO) regularly issues circulars and recommendations regarding the sea passenger industry for the simulation of procedures to evacuate ships [59], and the interaction between human evacuees and a physically constrained environment has been studied extensively in recent research [60,61,62].

Methods such as artificial potential fields and adaptive routing [63], possibly with partial reversal, have also been designed to assist evacuees in avoiding hazards. While many studies overlook the need to provide directions that guarantee that the “time to reach the exit” for each evacuee is below a specified upper bound that guarantees survivability, the AnLogic simulator [64] has explicitly incorporated the guarantee of an exit deadline for each evacuee in the system, as recommended for ship evacuation by the International Maritime Organization. The research in [65] has investigated the effects of imperfections in IoT systems, such as communication delays or message loss, as well as the effects of potential evacuee panic, on the performance of emergency evacuation procedures in cruise ships.

## 3. Modeling the Evacuation System as a Network of Queues

The evacuation system comprisesthe following:

- The *n* locations that evacuees enter or exit as they move from their entry point to the exit, which we call “nodes”;
- Corridors and staircases which connect the locations;
- Exit points which allow the evacuees to leave the vessel.

This structure is represented by a **directed acyclic graph** *G*, where the locations are the set of nodes V={1,…,n}, and directed arcs A(i,j) that represent the corridors, passageways, or staircases, and the direction of each arc represents the direction the evacuees take during an emergency evacuation. Any pair of nodes (i,j) can only be connected by a single directed arc A(i,j) from *i* to *j*, or A(j,i), but not both. If $A={\left[A\left(i,j\right)\right]}_{\left[n\times n\right]}$ is the binary matrix where A(i,j)=1 if and only if there is an arc from *i* to *j*, we have A(i,i)=0 and A(i,j).A(j,i)=0,∀i,j∈V.

*V* is composed of three disjoint subsets: the set of source nodes *S*, the set of exit (sink) nodes *F*, and the set of intermediate nodes *I*, such that V=S∪F∪I, where:

$$\begin{array}{ccc} S & = & \{i:\;such\;that\;A(j,i)=0,\;\forall \;j\neq i\},\\ F & = & \{i:\;such\;that\;A(i,j)=0,\;\forall j\neq i\},\\ I & = & \{i:\;such\;that\;i\notin S\cup F\}\;. \end{array}$$

This means that source nodes do not have predecessors, sink nodes do not have successors, while intermediate nodes have both successors and predecessors in the graph *G*. We also assume that *G* has an important property which is necessary for a graph that represents an evacuation system: each node is connected to at least one of the sinks by, at most, n−1 arcs. In other words, an evacuee can go from any node of *G* to a sink node by traversing, at most, n−1 directional arcs (which are staircases or passageways).

The AM we use to evaluate the total average delay for an evacuee, from any source to any exit point in the evacuation system, is a network of queues. We assume that each queue’s capacity is unbounded, which means that the corridors, passageways and staircases have been designed to be large enough so that they are never completely filled by people. We summarize the notation used in this paper in Nomenclature.

### The Arrival Rates of Evacuees into Corridors

We assume that each source node s∈S is a corridor $K_{s}$ which receives the evacuees exiting from individual cabins (or also possibly common rooms such as restaurants, lounges, etc.), according to independent, identically, and exponentially distributed inter-arrival times (a Poisson process) of rate $\lambda_{s}$. The evacuees traverse the corridor in a time of average value $\tau_{s}$, and enter at rate $\lambda_{s}$ into a waiting area $H_{s}$, where they queue in First-In–First-Out (FIFO) order. For a corridor *s* of length $L_{s}$, which contains $C_{s}$ cabins aligned along the sides of the corridor, where evacuees walk on average at *v* m/sec (meters per second), the average time it takes an evacuee to go from her cabin to the waiting point $H_{s}$ is $\tau_{s}=\;0.5\;L_{s}v^{-1}$.

Since there are $C_{s}$ cabins in the corridor $K_{s}$, and assuming that there are evacuees in each cabin and that evacuees exit cabins singly, we assume that the exit of evacuees into the corridor $K_{s}$ occurs as a Poisson process, i.e., with independent and exponentially distributed interarrival times of successive passengers:

(1) $$\lambda_{s}=\frac{C_{s}}{\tau_{s}}=\frac{2vC_{s}}{L_{s}}\;.$$

In the queueing model used in this paper, when A(i,j)=1, the staircase, passageway, or corridor K(i,j) that connects node *i* to node *j* for i∈S∪I and j∈I∪F is represented by a FIFO queue with independent and exponentially distributed service times of average value $\mu_{ij}^{-1}$. If j∈I, an evacuee leaving queue K(i,j) will enter another queue K(j,l) such that A(j,l)=1 with probability P(j,l) and $\sum_{v_{l}\in I\cup F}A\left(j,l\right)P\left(j,l\right)=1$. Finally, evacuees in $K_{f}$, f∈F, exit the system in FIFO order with an exit rate $\beta_{f}$, which is the parameter of an independent and identically distributed exponential service time at the final node.

## 4. Routing of Evacuees

In this section, we first describe the rules that will be compared for the routing of evacuees through the system after an overall “Exit” order has been given. The “Baseline Policy” considers that as soon as a passenger arrives at any node where she must select a passageway, she will not wait and directly move forward using one of the four Guidance Rules that are discussed below. On the other hand, we introduce the “Pull Policy,” where passengers exiting from their cabins or meeting rooms will then wait in “waiting areas” and access the evacuee system directed towards the exit in First-In–First-Out order only when they receive a message to move forward into the passageways, again using one of the Guidance Rules that are discussed below. The basic idea is that if evacuees are allowed to enter a passageway or staircase as soon as they arrive at its entrance, then this will add to the congestion at that staircase or passageway, so admission and routing rules are needed that can help address this issue.

The Pull Policy assumes that the message to move forward arrives (using wired or wireless communications) from an exit point at the end of the evacuation process, and that each of the waiting areas is selected at random with equal probability to receive the message. On the other hand, the “Pull Heuristic” will send the message to *all* of the waiting areas simultaneously, resulting in less waiting time in the waiting areas but possibly higher congestion.

We will compare four Guidance Rules to choose the subsequent link that an evacuee joins from any of the nodes in the evacuation system (except for the final exit node):

1. In “Guidance Rule 1,” an evacuee leaving a node *i* (except for the final exit node) selects its successor link K(i,j),j∈S(i) with equal probability, so no attention is paid to the relative speed of the links or to their congestion. The equal probability is expressed as $P\left(i,j\right)=\frac{1}{\left|S(i)\right|}$ provided that |S(i)|>0.
2. In “Guidance Rule 2,” an evacuee at a node (except for the final node) chooses its successor link K(i,c), which is the one that has the highest speed or service rate, i.e., the one that satisfies $\mu_{ic}\geq \mu_{ij},\;for\;j,c\in S\left(i\right)$. If there is more than one such link, then the one with the smallest value of *j* is chosen.
3. In “Guidance Rule 3,” each evacuee leaving some node *i* (except for the final node) selects a successor link K(i,c) with probability:
  (2) $$P\left(i,c\right)=\frac{\mu_{ic}}{\sum_{k\in S(i)}\mu_{ik}}.$$
  This Guidance Rule results in an interesting performance result that is detailed below in Section 4.1: the average delay of each of the shared downstream links, resulting from Guidance Rule 3, is identical for each of the links and corresponds to a link whose service rate is the average of all the link service rates and whose traffic rate is equal to $\lambda_{i}\times {\left(\left|S\left(i\right)\right|\right)}^{-1}$, i.e., an equally shared traffic rate over all of the links. Here, each node *i* enters into an “equivalent service center,” whose service rate is the average of the service rates of all the links that exit node *i* and whose incoming traffic rate is $\lambda_{i}$ divided by the number |S(i)| of outgoing links.
4. In “Guidance Rule 4,” evacuees at all nodes (except the exit nodes) select the path to the exit that minimizes the evacuees’ total time to the exit, verifying that the specified deadline to the exit is not violated for the worst-case link delays. Dijkstra’s well-known algorithm [66], or its variants [67], can be used to find the shortest path from a given source node to every other node in a graph or to find the shortest path to any specific destination node by stopping it when it finds the shortest path to some final node *f*. It is of complexity $O(n^{2})$ for *n* nodes.

In the specific case of cruise ship evacuation, the worst-case delay for each link is a conservative upper bound on the traversal time across the link, estimated based on the link length and the passenger walking speed on a cruise ship with a heel angle of 20 degrees. This walking speed is determined by the normal speed on a ship at an inclination angle of 0 degrees and by the direction of movement, as well as the speed reduction factor associated with a heel (or trim) angle of ±20 degrees. The selected deadline *D* is as follows:

(3) $$D=0.8\left(T_{S}-\frac{2}{3}T_{EL}\right)-T_{A},$$

where 0.8 is a safety factor, $T_{S}$ is the estimated survival time until capsizing for a cruise ship following an accident at sea, $T_{EL}$ is the time required for abandonment by all passengers and crew on board, and $T_{A}$ denotes the time taken by a passenger for all actions she takes after receiving the emergency notification until she begins moving. For instance, ccording to the IMO MSC.1/Circular.1533 [59] for the Yangtze Gold 7 cruise ship, $T_{S}=80$ min, $T_{EL}=30$ min, and A=10 min under night-time scenarios.

Note that each of these four rules uses decisions which are fixed a priori, and they do not require a computational or communication facility to be associated with individual nodes. However, the communication facility is indeed needed for the “Pull Policy” and the “Pull Heuristic” that are discussed below.

### 4.1. Key Property of Guidance Rule 3

With the assumption used in Guidance Rule 3, when the evacuees arrive to node *i* at rate $\lambda_{i}$, subsequently entering each link K(i,c) with probability P(i,c) in FIFO order, and assuming that the service time per evacuee is exponentially distributed with average value $\mu_{ic}^{-1}$, we know [35] that the average time spent by an evacuee in each link K(i,c),c∈S(i), is as follows:

(4) $$W_{ic}=\frac{1}{\mu_{ic}-\lambda_{i}P\left(i,c\right)},$$

so that the average time spent by an evacuee arriving at node *i* as she travels on the set S(i) of outgoing links from node *i* is as follows:

(5) $$\begin{array}{ccc} \sum_{c\in S(i)}P\left(i,c\right)W_{ic} & = & \sum_{k\in S(i)}\frac{1}{\frac{\mu_{ic}}{P(i,c)}-\lambda_{i}}=\sum_{k\in S(i)}\frac{1}{\sum_{k\in S(i)}\mu_{ik}-\lambda_{i}},\\ & = & \frac{1}{\frac{\sum_{k\in S(i)}\mu_{ik}}{\left|S(i)\right|}-\frac{\lambda_{i}}{\left|S(i)\right|}}\;. \end{array}$$

### 4.2. The Pull Policy

To further avoid congestion in passageways and staircases, one could also synchronize the entrance of evacuees into the passageways or staircases, with the departure of preceding evacuees from the end of the passageway. To do this, one would need a sensor at the exit of the passageway that detects the departure of an evacuee and a network-based communication system that sends a message to the entrance point of the passageway, where a visual display, e.g., a light that turns green or red, would limit or (to the contrary) encourage entrance into the passageway in a manner that offers congestion-free movement in that passageway.

In this paper, we consider a simpler approach commonly implemented in many practical settings for human services, which we call the Pull Policy, that requires communication between the exit point from the service system and the people in the waiting areas. One can actually observe this technology at work today, for customer or patient management, in various public service institutions such as hospitals and large administrative organizations that cater to the public.

For instance, in a large hospital, patients who arrive for consultations are first directed to a waiting area where they get a number from an automatic ticket machine and then sit and wait until a consultant physician or nurse becomes free. The departure of a patient from the consultation area triggers the «pull» of the next patient who is waiting in the area, in FIFO order, to the service area. When that happens, an electronic signpost displays the number of the patient being called and the number of the consultation room, and the patient whose number is called walks towards the designated consultation room.

The actual technology that is installed or used will depend on the available infrastructure, on the available budget, and on whether the evacuation process needs to be fully automatic or whether it should proceed under human control. For instance, the Pull Policy can also be implemented with SMS messaging on mobile phones. In a passenger ship, where there is a large technical crew, and the passengers are critical elements, we would expect that it would come under some partial human control to guarantee that every passenger is accounted for. However, we wish to stress that the Pull Policy does not require complicated technologies and is, in fact, currently used in service organizations as described above.

In an evacuation system, the Pull Policy can be described as follows:

- When an evacuee leaves an exit point *f* of the evacuation system, a message is sent with probability p(f,s) to all the waiting areas $H_{s}$ at the sources, with $\sum_{s\in S}p\left(f,s\right)=1$.
- If $H_{s}$ receives a message, and if it contains at least one evacuee, then the evacuee at the head of the waiting line in First-In-First-Out (FIFO) order at $H_{s}$, immediately enters into the corresponding passageway or staircase that is selected according to one of the “Guidance Rules 1–4”.
- At each $H_{s}$, there is also an “impatience time” of average value $\frac{1}{\alpha_{s}}$, after which an evacuee would be allowed into its preferred passageway or staircase even if a message is not received from the “exit” location *f*.
- We also evaluate the “Pull Heuristic” whose purpose is to reduce the chances that the message from the exit node arrives at waiting areas where there are no evacuees. Thus, in this case, the message from the exit point *f* is sent simultaneously to *all* waiting areas $H_{s}$, and is modelled heuristically by setting p(f,s)=1 for all sources *s*.

In the sequel we will use the term “Baseline Model” for the mathematical model that excludes the Pull Policy, or the Pull Heuristic, in which all nodes (except the final node) select their successor nodes with equal probability.

### 4.3. Analytical Solution

The analytical solution of this network of queues is a special case of the “G-Networks with triggered customer movement” introduced in [35]. Based on this analytical solution, we obtain the following results. We recall that $Q_{f},\;0\leq Q_{f}<1$, is the probability that a final node f∈F has at least one evacuee waiting to exit the evacuation system, and $Q_{s}$, $0\leq Q_{s}<1$, is the steady-state probability that a waiting space $H_{s},s\in S$, has at least one evacuee waiting in $H_{s}$ to enter the evacuation system. We then have

(6) $$Q_{s}=\frac{\lambda_{s}}{\alpha_{s}+\sum_{f\in F}Q_{f}\beta_{f}p\left(f,s\right)},\;s\in S.$$

We already know $\lambda_{s},\;s\in S$ from (1), but we also have to solve linear equations for the rate of flow of passengers, in NPS, into each of the other nodes:

(7) $$\begin{array}{ccc} \lambda_{i} & = & \sum_{s\in S}1\left[s=i\right]\lambda_{s}+\sum_{l\in I}\lambda_{l}A\left(l,i\right)P\left(l,i\right),\;i,l\in I\; \end{array}$$

(8) $$\begin{array}{ccc} \lambda_{f} & = & \sum_{s\in S}\lambda_{s}A\left(s,f\right)P\left(s,f\right)+\sum_{l\in I}\lambda_{l}A\left(l,f\right)P\left(l,f\right),\;f\in F,\; \end{array}$$

yielding $\lambda_{i},\;\lambda_{l},\;\lambda_{f}$. This then allows us to compute

(9) $$q_{ij}=\frac{\lambda_{i}P\left(i,j\right)}{\mu_{ij}},\;Q_{f}=\frac{\lambda_{f}}{\beta_{f}}.$$

Let *N* denote the average of the total number of evacuees in the evacuation system in steady-state; it is obtained from the previously defined quantities, as follows:

(10) $$N=\sum_{s\in S}\left(\tau_{s}+\frac{Q_{s}}{1-Q_{s}}\right)+\sum_{f\in F}\frac{Q_{f}}{1-Q_{f}}+\sum_{i=1,j=1,i\neq j}^{n}A\left(i,j\right)\frac{q_{ij}}{1-q_{ij}}\;,$$

and the total average time spent by an evacuee in the evacuation system, denoted by *W*, can be derived using Little’s Law [68,69]:

(11) $$W=\frac{N}{\lambda },\;where\;\lambda =\sum_{s\in S}\lambda_{s}\;.$$

#### Scalability Issues

Since the values of $\lambda_{s},\;s\in S$ are computed from (1) in a total of |S| steps, the key remaining computation is to solve the n−|S| **linear** Equations (7) and (8). Since the graph *G* is acyclic, it follows that the system of Equations (7) and (8) can be solved with a number of operations of order $O((n-{\left|S|\right)}^{2})$. On the other hand, since we need to store all the connectivity information for *G*, the space complexity for solving the model is $O(n^{2})$. So the manner in which computation time and memory space varies with the size *n* of the system is well understood and corresponds to a case of low polynomial complexity that can be solved with a conventional linear equation solver.

Let us also point out that for the Pull Policy, the number of messages needed (amount of communication) from any single exit point is only one message per exiting passenger. On the other hand, the Pull Heuristic with |S| waiting areas will send |S| messages for each passenger that exits the system.

## 5. Numerical Results

**Figure 4:**
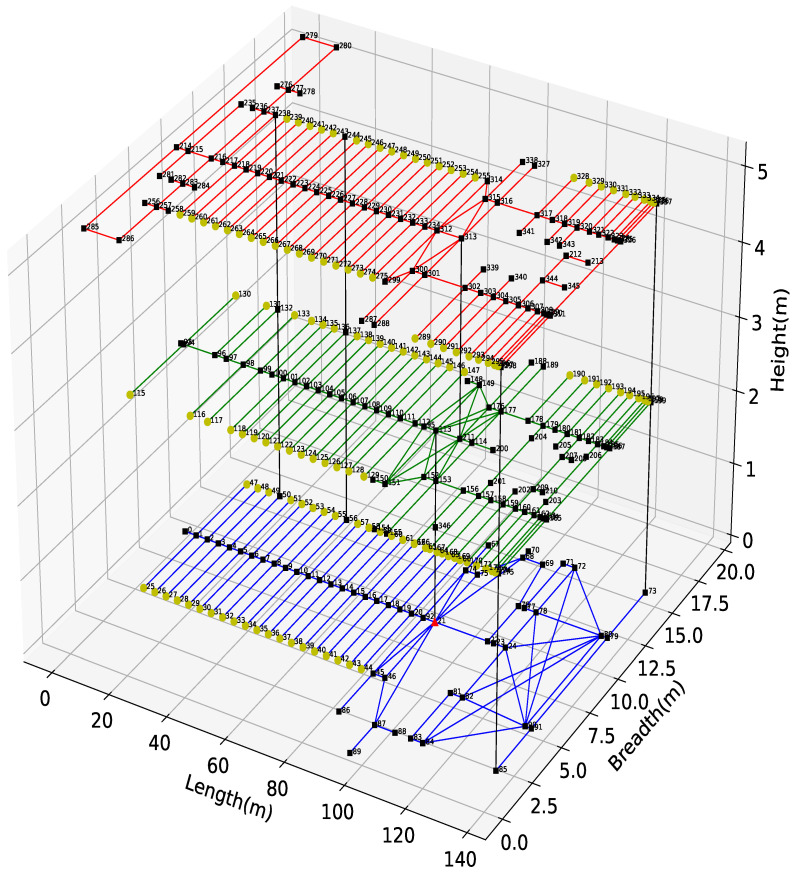
This figure shows a graph model of the emergency exit system that is being simulated. Here, the blue, green, and red solid lines designate the passageways on the second, third, and fourth floors, respectively, of the ship. The black solid lines represent the staircases connecting the different floors. The single exit node is marked with a RED triangle. The cabins are shown with light green circles. The black squares designate the nodes that represent other locations (such as lounges and restaurants) where passengers may congregate, as well as waiting areas, locations that connect passageways to each other and that connect staircases and passageways. The black numbers next to the nodes indicate their unique identifiers in the graph model.

The environment we use for the numerical results that are presented in this paper, encompasses the second, third, and fourth floors of the Yangtze Gold 7 cruise ship shown in Figure 4 as a graph consisting of 528 edges and 347 nodes. The blue, green, and red solid lines represent the passageways on the second, third, and fourth floors, respectively, while black solid lines denote the staircases connecting the different floors. There is a single exit node which is marked with a red triangle, while the nodes representing the cabins are labelled with light greencircles, and the remaining nodes are shown as black squares. Additionally, the black numbers next to the nodes indicate their unique identifiers.

The typical delay across each edge is estimated based on its length as well as the average walking speed of evacuees on a cruise ship under normal conditions. In our simulation, according to IMO MSC.1/Circular.1533 [59], the average walking speed on flat terrain (e.g., corridors) is set to 0.56 m/s, while the speeds for going downstairs and upstairs are set to 0.45 m/s and 0.37 m/s, respectively. In Table 1, we summarize the key parameters concerning the source nodes and the exit.

### 5.1. Evaluation of the Pull Policy

**Figure 5:**
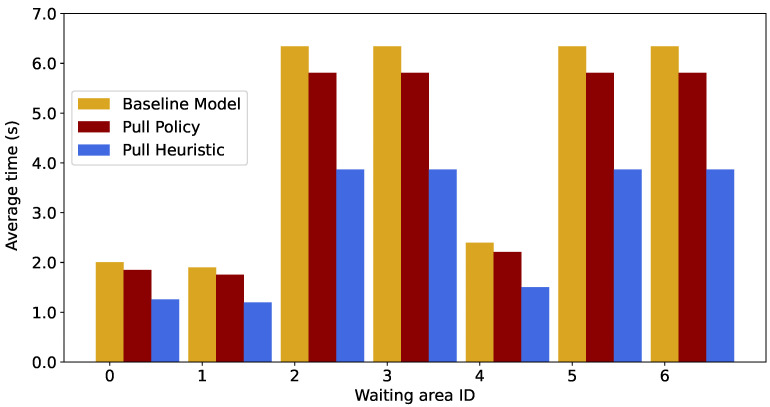
Comparison of the average time spent in each waiting area under “Guidance Rule 1” across the three algorithms when $\alpha_{s}=0.035$ and λ=0.02.

We compare the average time spent in each waiting area using the pull algorithm against the two aforementioned algorithms, based on “Guidance Rule 1,” as shown in Figure 5. The results under the other three Guidance Rules are identical to those of “Guidance Rule 1.” The service rate at the waiting areas is fixed at $\frac{1}{16}\beta_{f}$ (i.e., 0.035). The total arrival rate at the source nodes is set to 0.02.

It can be observed that the absence of messages from the output node *f* leads to a deterioration of evacuation performance, as indicated by the increased average time spent by evacuees across all waiting areas. Moreover, the Pull Heuristic algorithm results in a shorter average time spent in the waiting areas compared with the standard Pull Policy due to the higher probability that messages from *f* are received by the waiting areas.

**Figure 6:**
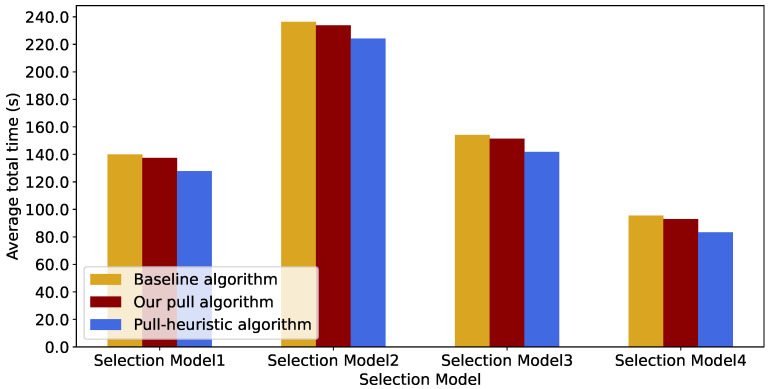
Comparison of the average total time spent in the system with the three algorithms under all four Guidance Rules when $\alpha_{s}=0.035$ and λ=0.02.

To assess the impact of the messages transmitted from the exit to the waiting areas on overall evacuation efficiency, we compare the average total time spent in the system, calculated using the three algorithms across all four Guidance Rules. As illustrated in Figure 6, the reception of messages by the waiting areas improves evacuation efficiency across all four Guidance Rules. That is, both the pull algorithm and the pull heuristic algorithm show a reduction with respect to the average total time spent by evacuees in the system. In addition, it is worth noticing that “Guidance Rule 4” consistently yields a shorter average total time compared to the other three Guidance Rules with all three algorithms under $\alpha_{s}=0.035$ and λ=0.02. This is mainly because in this group of simulations, evacuees are subject to a mild congestion due to the low total arrival rate at the source nodes. The successor passageway selections made by “Guidance Rule 4” can achieve the shortest typical traversal time to the exit with regard to a specified deadline bound *D*, provided that waiting times caused by potential crowd congestion are not taken into consideration.

Figure 6 simply confirms that Guidance Rule 4 minimizes the average total evacuation time when there is no congestion, i.e., provided that the total arrival rate at the source nodes is very low, as compared to the other Guidance Rules.

### 5.2. Impact of the Total Arrival Rate at Source Nodes

In practical emergency evacuation scenarios aboard cruise ships, the number of evacuees that occupy different cabins can vary, which causes different arrival rates at the source nodes. Thus, in this section, we examine the influence of the total arrival rate at source nodes on evacuation efficiency measured by the average total time spent within the system. We run the three algorithms across all four Guidance Rules with different preconfigured total arrival rates at source nodes (0.02,0.04,0.06,0.08,0.1,0.12,0.14,0.16,0.18). When λ = 0.2, the system becomes unstable across all three algorithms under each of the four Guidance Rules. In this group of simulations, the evacuee average waiting time at the waiting areas is set to eight times the average inter-exit time to be large enough so as not to influence the results; evacuees only very infrequently move downstream without the arrival of a message from the exit node $\alpha_{s}=0.125\beta_{f}$.

**Figure 7:**
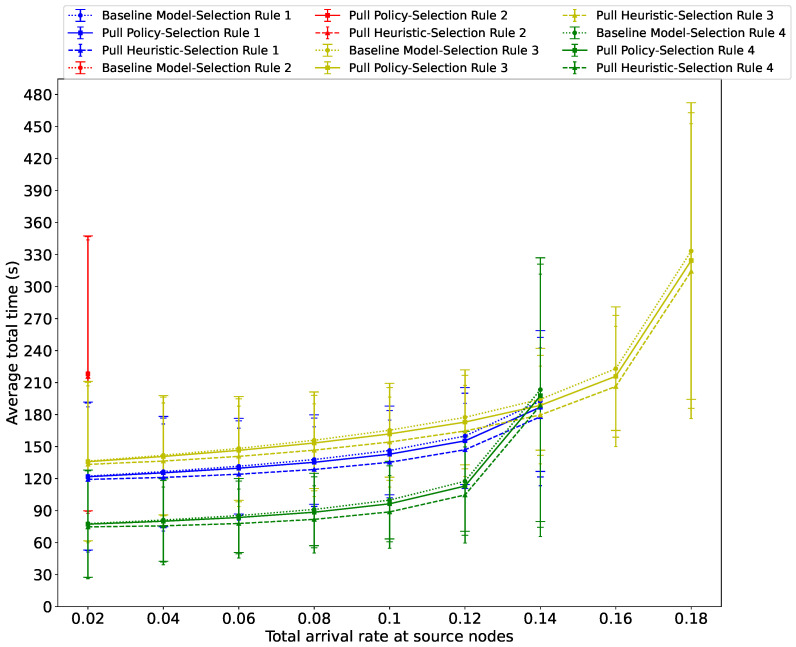
Average total time spent by evacuees in the system versus the total passenger arrival rate λ at the source nodes, using the three algorithms across all four Guidance Rules when $\alpha_{s}=0.07$.

Figure 7 presents the average total time spent by evacuees in the system, including the time at the source nodes, the intermediate links, and the exit, as a function of the total passenger arrival rate λ at the source nodes, and we see that it increases, culminating to a critical threshold beyond which the system saturates. Note that the threshold values of λ where saturation occurs will differ across the four Guidance Rules:

- Under Guidance Rule 2, the system becomes unstable when λ reaches 0.04, regardless of the algorithm employed, due to congestion on link (80, 78), where 80 and 78 denote the IDs of two intermediate nodes, as illustrated in Figure 4.
- We also observe that the λ value under which the system reaches saturation under Guidance Rule 4 is lower compared to the saturation level when Guidance Rule 3 is used. This is because evacuees under Guidance Rule 2 will choose the successor link with the highest service rate, while under Guidance Rule 4, they select the successor link that yields the shortest typical travel time to the exit for a given deadline *D*. In contrast, under Guidance Rule 3, because of the smart probabilistic traffic sharing, the system remains stable at higher total arrival rates at source nodes, compared with Guidance Rule 2 and Guidance Rule 4.
- Moreover, we can see that when the total arrival rate at source nodes is below 0.14, the total average evacuation delay with Guidance Rule 4 is consistently the smallest among all four Guidance Rules and across all three algorithms.
- When the total arrival rate at source nodes reaches or exceeds 0.14, severe congestion is observed along the path with the shortest typical travel time for a specified deadline *D* across all three algorithms. This is observed in Figure 7 from the average total exit times for the four Guidance Rules under all three algorithms. Furthermore, Guidance Rule 1 consistently achieves the best performance at λ=0.14 across the three algorithms, whereas Guidance Rule 3 outperforms the other three Guidance Rules when λ exceeds this value.

Last but not least, our results show that the Pull Policy and the Pull Heuristic reduce the average total time that the evacuees spend in the evacuation system under all of the Guidance Rules for all values of λ where stability is preserved.

**Figure 8:**
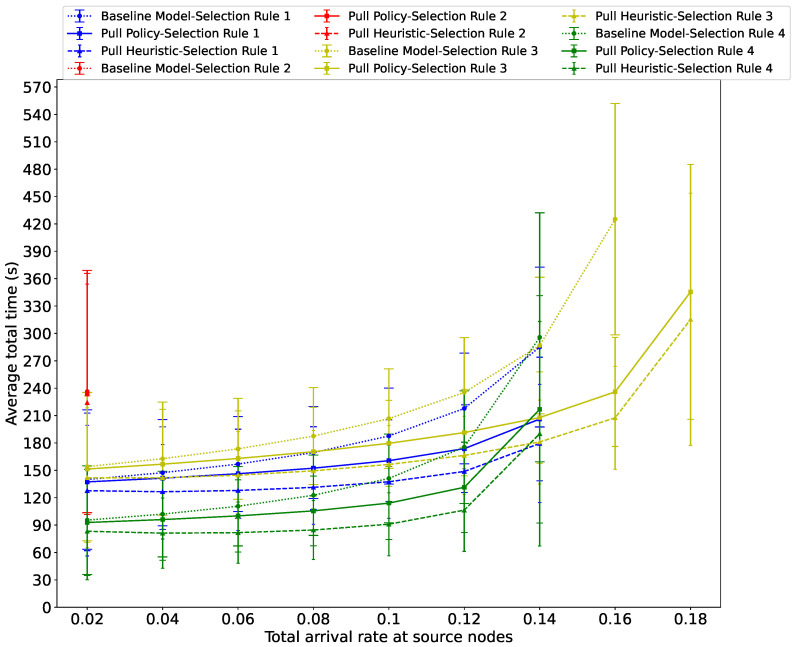
Average total time spent by evacuees in the system versus the total passenger arrival rate at the source nodes using all three algorithms across the four Guidance Rules when $\alpha_{s}=0.035$.

To compare the performance of the three algorithms with varying service rates at the waiting areas, in Figure 8, we present the average total time across various total arrival rates at source nodes, with $\alpha_{s}$ set to 0.035 (i.e., $\alpha_{s}=\frac{1}{16}\beta_{f}$). We observe that under “Guidance Rule 3,” the system becomes unstable when the total arrival rate at source nodes exceeds 0.16 with the Baseline Algorithm, while the other two Guidance Rules preserve stability when higher values of λ are used. The waiting area located in the second source node on the third floor (i.e., $s_{32}$) is the primary contributor to this transition of the system to instability under the Baseline Algorithm combined with Guidance Rule 3. Again, we see that the use of messages sent from the exit to the waiting areas when evacuees leave the system is useful to preserve the system stability under higher total arrival rates at the source nodes.

### 5.3. Impact of Service Rate at Waiting Areas

In order to assess the impact of service rates at the waiting areas on evacuation efficiency, we carry out several numerical experiments to compare the average total time experienced by evacuees in the system under all four Guidance Rules across the three algorithms for varying values of $\alpha_{s}$. The service rate at the waiting areas, $\alpha_{s}$, ranges from $\frac{1}{16}\beta_{f}$ (i.e., 0.035) to $\frac{1}{2}\beta_{f}$ (i.e., 0.28). In the controlled simulation, $\alpha_{s}$ is capped at $\frac{1}{2}\beta_{f}$, considering that excessively high values of $\alpha_{s}$ may induce congestion in intermediate links or at the exit, due to burst inflows from the waiting areas into the system.

**Figure 9:**
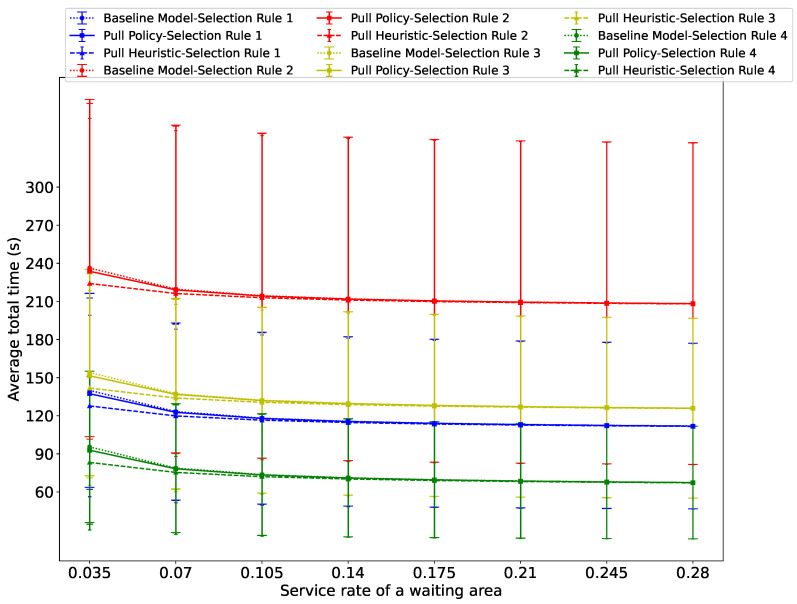
Average total time spent by evacuees in the system versus the service rate at the waiting area when the passenger arrival rate is λ=0.02.

**Figure 10:**
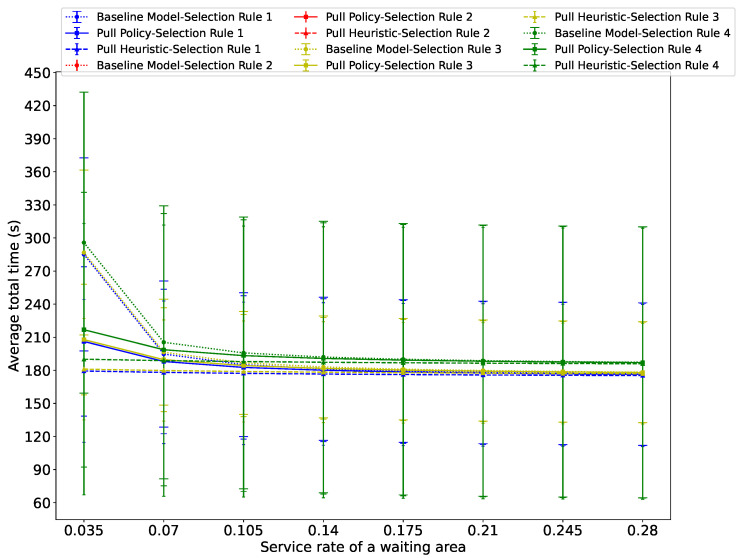
The average total time spent by evacuees in the system versus the service rate at the waiting area when the passenger arrival rate is λ=0.14. We see that with Guidance Rule 2, the system saturates with all three algorithms, and that the total average evacuation time with Guidance Rule 1 is close to that of Guidance Rule 3 and smaller than with Guidance Rule 4, for all three algorithms, due to the reduced congestion of probabilistic load sharing with Guidance Rules 1 and 2.

**Figure 11:**
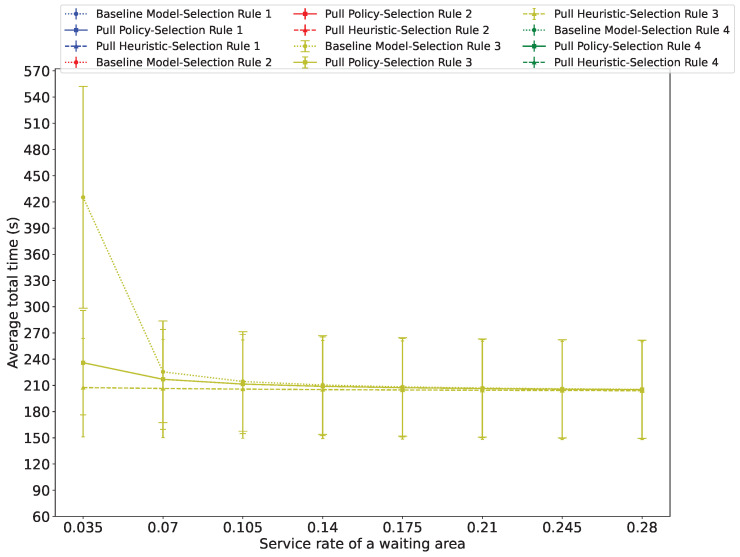
The verage total time spent by evacuees in the system versus the service rate at the waiting area when the passenger arrival rate is λ=0.16. This figure only shows the curves obtained with Guidance Rule 3, while the system is unstable with the other three Guidance Rules, demonstrating the value of probabilistic smart load sharing by Guidance Rule 3.

Figure 9, Figure 10 and Figure 11 show the average total evacuation time as a function of the service rate at the waiting areas, for three distinct total arrival rates at the source nodes: λ=0.02, λ=0.14, and λ=0.16. The figures show that in all cases, the average total time decreases as $\alpha_{s}$ increases, albeit at a progressively decelerating rate.

Figure 9 shows that Guidance Rule 4 achieves the best performance our of all four Selection Rules across the Baseline, Pull Policy, and Pull Heuristic algorithms when λ=0.02, mainly due to the ability of Guidance Rule 4 to identify the fastest evacuation route for a given deadline *D* under low total arrival rates at the source nodes, when congestion does not occur on the routes that are chosen.

However, when the total arrival rate at the source nodes is 0.14, Figure 10 shows that with Guidance Rule 2, the system saturates with all three algorithms. Also, the total average evacuation time with Guidance Rule 1 is close to that of Guidance Rule 3 and smaller than that of Guidance Rule 4 with all three algorithms, primarily due to the reduced congestion thanks to the probabilistic load sharing used by Guidance Rules 1 and 2.

When the total arrival rate is λ=0.16, Figure 11 shows the curves obtained with Guidance Rule 3, while the system is unstable with the other three Guidance Rules, highlighting the value of the probabilistic smart load sharing offered by Guidance Rule 3. The results in Figure 11 also clearly show the effect of messages from the exit to the waiting areas. The average total exit time of the evacuees decreases significantly when the Pull Policy or the Pull Heuristic are used, confirming the results presented in Figure 9 and Figure 10.

## 6. Conclusions and Further Research

In the emergency evacuation of ships, as well as other vehicles such as aircraft, safe and reliable methods are needed to ensure the fast movement of passengers and evacuees towards the most appropriate exits. Thus, many large vehicles, such as cruise ships, rely on prior planning to instruct the evacuees as to the best course of action when an emergency occurs.

Because of the human consequences and high costs of hazards and accidents that may require an evacuation process to be carried out, substantial research has been conducted to investigate the design of technology-assisted methods to provide evacuees with the best advice regarding evacuation procedures during emergency events. However, such systems can require expensive installations that must be built into the cruise ship, and which will require constant maintenance and updates in order to remain effective.

Thus, we introduce, discuss, and evaluate several useful algorithms that can reduce congestion and increase the speed with which evacuation can be conducted safely. To this end, we consider two aspects of the evacuation process that can minimize evacuation times from a built structure or large transport vehicle, such as a ship. The first approach involves reducing the average total evacuation times through smart evacuation procedures that distribute the evacuees over multiple paths and take full advantage of the available capacity of each path simultaneously. The second approach, which can be used together with the previous one, is the Pull Policy, which synchronizes the access of the evacuees to the evacuation paths following the safe exit of the preceding evacuees from the system such that bottlenecks and congestion may be avoided. The Pull Policy moves one passenger from a single waiting area that is chosen at random among all the waiting areas for every passenger that exits the system. We also evaluate the Pull Heuristic, which pulls one passenger from each of the waiting areas towards the exit each time a passenger leaves the system.

This paper then evaluates these approaches using a queueing network model that allows for fast, computationally efficient solutions, and ease in varying the parameter values very widely. This offers the system designer a means by which to compare the approaches with a baseline and with each other and to do so very rapidly at low computational cost. Conventional analytically tractable queueing models do not allow for the incorporation of state-dependent control actions, and a state-dependent control action is at the heart of the Pull Policy that we discuss in this paper, where each passenger in a waiting area is “pulled” to continue the evacuation following the departure of a passenger through an exit point. This is why we use a queueing networks generalization, known as G-Networks, which allows for the computationally tractable modeling of such control actions. We note that the solution to the resulting equations has a computational complexity of $O(n^{3})$ when there are *n* locations if the network model has feedback loops, and a computational complexity of $O(n^{2})$ when it is a feedforward network. In the present case, there are no feedback loops in the passengers’ paths; hence, we have a computational complexity of $O(n^{2})$, while the amount of memory space used is of order $O(n^{2})$.

The analysis we conducted compares the various relevant routing techniques for evacuees, providing useful output in terms of response time curves as a function of the relevant parameters, including the arrival rate of passengers to the system, the number of evacuees being handled per unit time, and the values of various system parameters such as the passenger traversal speeds. These speeds are known on average, but the use of a probability model allows for a very wide variation in individual values for a given collective average value, including for the average waiting time $1/\alpha_{s}$ at any waiting area *s*, the arrival rates of passengers to the evacuation system α, the average walking speeds in the passageways and staircases, and the average speed $\beta_{f}$ at which passengers leave the final exit point. This paper shows the outcomes of the various policies that we propose with respect to a realistic use-case: the Yangtze Grand ship evacuation system. However, there are no limitations to the use of the Pull Policy (and the Pull Heuristic) in other physical layouts or contexts. The only requirement is the need for communication between the exit points and the passenger waiting areas.

In future research, we plan to examine more detailed controls that can avoid congestion while maintaining a maximum throughput of evacuees through IoT-based signaling and control, employing adaptive schemes that react to bottlenecks before they occur. We will also investigate the possible effect of signaling and communication failures and suggest architectures that are able to react efficiently, maintaining a robust level of performance in the presence of such failures.

## Figures and Tables

**Table 1:** Parameters regarding the source nodes and the exit node.

Source/Exit Node	Corridor Length	No. of Cabins	Avg. Inter-Exit Time
**Node ID**	$L_{s}$ **Meters**	**Number $C_{s}$**	**Final Node** f: $\beta_{f}^{-1}$
Source21	76	38	
Source31	80	31	
Source32	26	9	
Source33	26	9	
Source41	64	33	-
Source42	26	9	-
Source43	26	9	
Exit21			1.786

## Data Availability

The data presented in this study are available on request from the authors. The data are not publicly available due to privacy or ethical restrictions.

## Nomenclature

The following abbreviations are used in this manuscript:

**Notation**	**Meaning**
*D*	Deadline or recommended maximum traversal time from a cabin to the system exit
$T_{S}$	Estimated time to capsize following a ship accident
$T_{A}$	The awareness time after emergency notification
$T_{EL}$	Time required for embarkation and launching
*G*	Directed acyclic graph representing the evacuation system
*V*	The set of all nodes in *G*
*n*	The number of nodes in *G*
*A*	The n×n binary adjacency matrix with elements A(i,j),i,j∈V
*S*	Set of source nodes, S⊂V, that correspond to corridors leading to waiting areas
*F*	Set of exit nodes, F⊂V
*I*	Set of intermediate nodes, I⊂V
$K_{s}$	Corridor receiving evacuees from the cabins to waiting area $H_{s},\;s\in S$
$L_{s}$	The length in meters of corridor $K_{s}$
$C_{s}$	The number of cabins along the sides of corridor $K_{s}$
$\tau_{s}$	Average traversal time of a passenger through corridor $K_{s}$ in seconds
$H_{s}$	Waiting area at the end of the corridor $K_{s},s\in S$
*v*	Average walking speed (meters/sec) of a passenger in a corridor
NPS	Number of Passengers per Second
$\lambda_{s}$	Average arrival rate in NPS, to $H_{s}$
λ	Total average arrival rate in NPS, to all the waiting areas
$\lambda_{i}$	Average arrival rate of evacuees in NPS into node i∈I
$\lambda_{f}$	Average arrival rate of evacuees in NPS into exit f∈F
K(i,j)	Corridor, passageway, or staircase connecting nodes i∈S∪I to j∈I∪F
$\lambda_{i}$	Average arrival rate of evacuees in NPS into node *i*
$\mu_{ij}$	Average departure rate in NPS from K(i,j) when it contains at least one passenger
P(j,l)	The probability of entering K(j,l) from $H_{j}$ or from K(i,j),j∈S∪I,l∈I∪F
$K_{f}$	The passageway through which evacuees leave the system at exit point f∈F
$\beta_{f}$	The average exit rate in NPS from $K_{f}$, when it is busy with at least one passenger
$W_{ij}$	Average time in seconds that an evacuee spends in $K_{ij}$
p(f,s)	Probability that a message from *f* is sent to $H_{s}$ in the Pull Policy and Pull Heuristic
$\alpha_{s}$	Average impatience rate in NPS of the passenger at the head of the queue at $H_{s}$
$Q_{s}$	The steady-state probability of having at least one evacuee at $H_{s}$
$Q_{f}$	The steady-state probability that $K_{f}$ contains at least one evacuee
$q_{ij}$	The steady-state probability that K(i,j) contains at least one evacuee
*N*	The average total number of evacuees in the system
*W*	Average total time in seconds spent by evacuees in the system
